# Comparison of Trotting Stance Detection Methods from an Inertial Measurement Unit Mounted on the Horse’s Limb

**DOI:** 10.3390/s20102983

**Published:** 2020-05-25

**Authors:** Marie Sapone, Pauline Martin, Khalil Ben Mansour, Henry Château, Frédéric Marin

**Affiliations:** 1 Université de Technologie de Compiègne, Alliance Sorbonne Université, UMR CNRS 7338 BioMécanique et BioIngénierie, 60200 Compiègne, France; khalil.ben-mansour@utc.fr (K.B.M.) ; frederic.marin@utc.fr (F.M.); 2 Ecole Nationale Vétérinaire d’Alfort, USC INRAE-ENVA 957 BPLC, CWD-VetLab, 94700 Maisons-Alfort, France; pmartin@lim-group.com (P.M.) ; henry.chateau@vet-alfort.fr (H.C.); 3 LIM France, Chemin Fontaine de Fanny, 24300 Nontron, France

**Keywords:** horse, locomotion, gait events, biomechanics, inertial measurement units, methods comparison, stride segmentation

## Abstract

The development of on-board sensors, such as inertial measurement units (IMU), has made it possible to develop new methods for analyzing horse locomotion to detect lameness. The detection of spatiotemporal events is one of the keystones in the analysis of horse locomotion. This study assesses the performance of four methods for detecting *Foot on* and *Foot off events.* They were developed from an IMU positioned on the canon bone of eight horses during trotting recording on a treadmill and compared to a standard gold method based on motion capture. These methods are based on accelerometer and gyroscope data and use either thresholding or wavelets to detect stride events. The two methods developed from gyroscopic data showed more precision than those developed from accelerometric data with a bias less than 0.6% of stride duration for *Foot on* and 0.1% of stride duration for *Foot off*. The gyroscope is less impacted by the different patterns of strides, specific to each horse. To conclude, methods using the gyroscope present the potential of further developments to investigate the effects of different gait paces and ground types in the analysis of horse locomotion.

## 1. Introduction

The analysis of the locomotion is an essential point for the monitoring of the physical integrity of the sport horse [1]. During locomotion, the distal limbs of the horse behave like a spring-mass system that stores energy during the damping phase when the hoof is down on the ground and restores it during the propulsion phase [2]. Consequently, the anatomical structures of the horse′s limb are subjected to high mechanical stress during locomotion [3]. In addition, mechanical stress could be emphasized by the nature of the ground [4] or the movement performed [5] i.e., landing after jumping a vertical fence [6]. These mechanical stresses repeated during the sporting exercise can lead to micro-lesions possibly leading to more serious injury causing lameness [7]. In the worst case, this leads to large veterinary costs and convalescence for the horse.

Head and pelvis movements are the most common criteria in locomotion analysis for the detection and localization of lameness in horses [8,9,10]. These movements need to be analyzed in conjunction with accurate detection of the left and right stance phases. This study is a preliminary step to the development of an on-board tool for the detection of degradation of horse locomotion. For this purpose, easy to use equipment is needed in order to make it useable in the field. The horse boots being a commonly used equipment to protect the horse limbs, we chose to explore the possibility to measure gait events at the level of the boots.

The identification of gait events in human locomotion based on inertial measurement units (IMU) data is currently a vivid research area [11,12]. Several methods are proposed using the accelerometer data [13,14] or the gyrometer data [14,15] based on threshold [16] or signal pattern identification [17]. However, each method presented advantages and limitations, and necessary direct comparison would help to define its informed applications. Horse locomotion being very different from human locomotion [18], the use of IMUs for horse locomotion analysis requires the development of specific methods.

The detection of spatiotemporal events, i.e., *Foot on* and *Foot off* timings, is an essential step for data preprocessing in order to segment the data and then calculate the locomotor parameters. This step is the keystone for the analysis of the locomotion of the horse [19], especially for the next detection of lameness [20]. Different methods have been explored to detect these events by force plate [21,22,23] or kinematic recordings [24,25,26]. In laboratory conditions, these methods are reliable but require specific equipment such as a force plate or a full motion capture (MoCap) system. New versatile methods based on the use of inertial measurement units (IMUs) allow on-board recordings of locomotion and in-field sessions [27,28]. 

Due to the fact that IMU records accelerations and rotational velocities, signal processing is required to detect specific spatiotemporal events of the horse locomotion. Signal processing methods, based on the coupled use of several IMUs on the horse, have been the subject of several studies [20,27,28,29]. These studies have provided methods for the precise detection of stride events from the combined use of IMUs positioned on the trunk and distal limbs [20]. The combined use of eight IMUs on specific anatomical areas has allowed the analysis of several locomotor parameters important for the analysis of locomotion and the quantification of lameness [27]. Other studies have developed methods of locomotion analysis from a single sensor, with, for example, the use of a geometric model to estimate the movements of the tuber coxae from an IMU positioned on the sacrum [28] or the use of an accelerometer positioned on the hoof [29]. This last study showed artefacts in the measurements which limits its usefulness in the precise evaluation of the locomotion of the horse. Few studies have focused on the development of the locomotion analysis method from IMU positioned only on the distal part of the limbs [30,31]. The first study [30] is based on the analysis of the symmetry of the gait at each stride cycle but did not detect the precise events delimiting the stance phase. The second study [31] evaluated the performance of four methods developed from signals of an IMU positioned on the distal horse’s limb to detect the two events delimiting the stance phase, named *Foot on* and *Foot off*. Despite the encouraging performance of this study, the explanation of the methods used was not fully detailed. 

The objective of this study is to explore different methods of detecting *Foot on* and *Foot off* events in order to increase the precision of on-board measurements of stride events. Four methods were developed from the signals of an IMU positioned on the distal front limb and compared using a reference MoCap system to assess accuracy. 

## 2. Materials and Methods

### 2.1. Animals

Eight sound horses of trotting breeds (four geldings and four mares, height 162 ± 3 cm (mean ± sd)) from the “Centre d’Imagerie et de Recherche sur les Affections Locomotrices Equines” (CIRALE) were included in the study. Prior to the procedure, the protocol was examined and approved by the dedicated ethics committee on animal investigation (Comité National de Reflexion Ethique sur l’Experimentation Animale, Anses/ENVA/UPEC n°HE_2017_01). 

### 2.2. Data Acquisition

**Figure 1:**
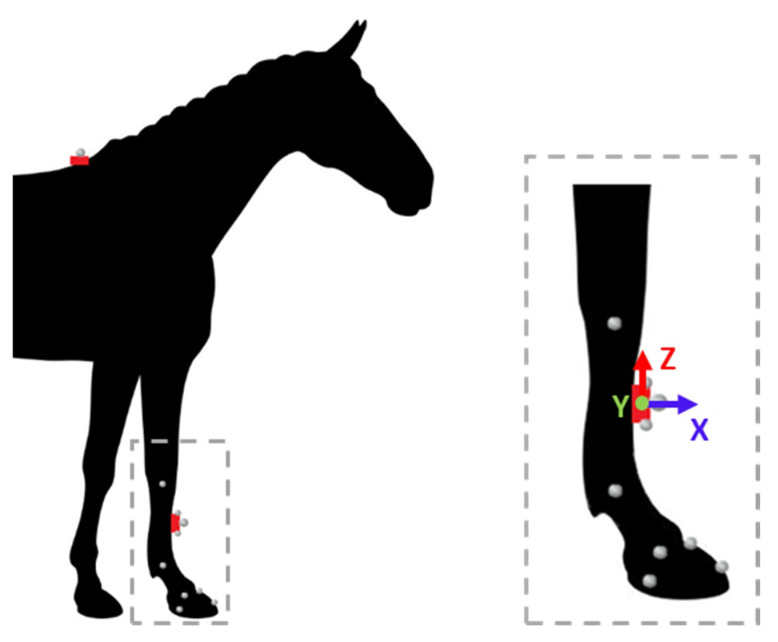
Placement of the two IMUs (represented in red) and the kinematics markers according to points of interest: six at anatomical points: carpal joint, metacarpo-phalangeal joint, hoof (toe, heel, front coronary band, lateral coronary band), one at the center of wither’s IMU and three on canon bone’s IMU (center, up lateral part, down lateral part). One additional free marker was used for synchronization keystroke on the wither’s marker.

**Figure 2:**
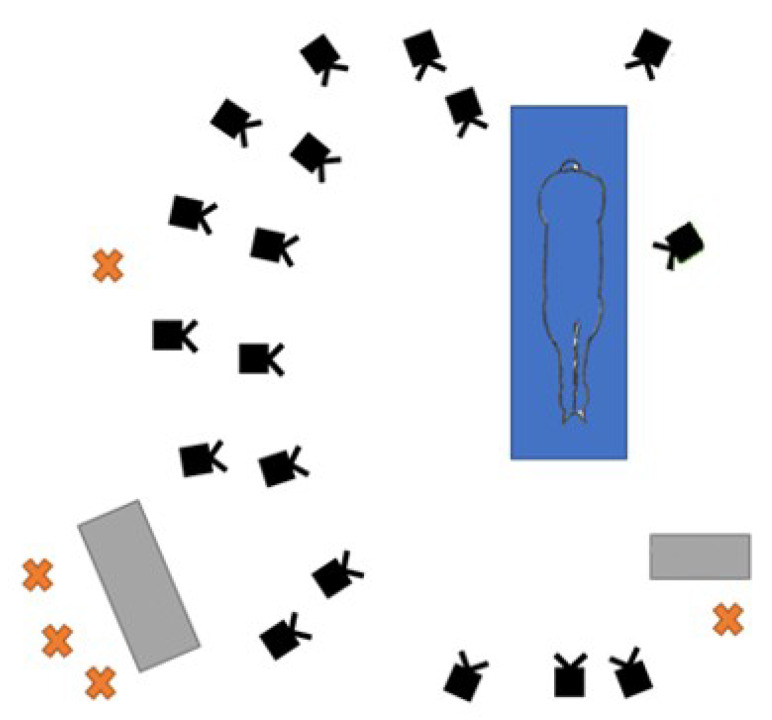
Positioning of Vicon cameras (in black) around the treadmill (in blue) to record the locomotion of the limbs of the right side of the horses. The orange crosses represent the different experimenters and their control tables (computers with software for MoCap and IMUs and treadmill control panel) are shown in gray.

Data acquisition included motion capture sessions with a marker-based system and IMUs (Figure 1). Motion capture was used to track horse limbs kinematics by 3D displacements of anatomical markers and marker clusters. Eighteen cameras (Vicon T160, Oxford Metrics Ltd., Oxford, UK) at full resolution 4704 × 1728, sampling at 200 Hz, were set up on both sides of a high-speed treadmill (Protrainer, Hippocenter, Saint-Paul du Verney, France) (Figure 2). Eight horses were equipped with 10 reflective kinematic markers, with a radius of 5 mm and 10 mm (only the 2 markers positioned in the center of each IMU) on the right forelimb and withers. The size of the mocap’s marker is a compromise to determine the smallest size possible allowing the optical detection, the 3D tracking, and risk of removal according to the high acceleration of the horse’s limb during locomotion. Two IMUs (ProMove-mini, Inertia Technology BV, Enschede, The Netherlands) with a full-scale range of 16 g, 2000 °/s, 16 bits, sampling at 500 Hz, were also positioned, for the first one, on the distal front limb of the horse, in the center of the dorsal side of the third metacarpal bone, and for the second, on the wither, used for system synchronization. The positioning of the IMU of the distal limb was carried out so as to align the Y-axis of the gyroscope with the axis transverse to the canon bone. The recordings of the two IMUs are managed by the gateway Inertia which ensures the synchronization (offset <100 ns) between the two sensors. Each horse performed three trot passes on a treadmill at a speed of 4 m/s, which is a usual speed at which horses are observed during clinical examination [32,33]. At least 25 strides were recorded at a steady speed for each trial. To synchronize the marker-based MoCap system with the IMU system, an additional kinematic marker was used to strike the IMU marker positioned on the wither at the beginning and at the end of each trial.

### 2.3. Data Processing

Data processing included five steps: (1) the raw data management, (2) the synchronization between the marker-based and IMU motion data, (3) marker-based data processing, (4) IMU data processing, and (5) statistical analysis. 

First, the raw measurements of both motion-capture systems were managed. From the marker-based system, 3D coordinates for each marker named MoCap data were computed by the manufacturer’s dedicated software (Nexus 2.8.0, Oxford Metrics Ltd., Oxford, UK). From IMUs, 3D accelerations and rotational velocities, named IMU data, were extracted from the dedicated software (InertiaStudio, Inertia Technology BV, Enschede, The Netherlands). Then MoCap and IMU data were processed by calculation software (Matlab R2019b, The MathWorks Inc., Natick, MA, USA). 

Second, data from the Mocap system and IMU were homogenized at 500 Hz using a continuous derivative interpolation function. Once the sampling frequency was standardized, a synchronization process has been performed. On the data coming from the IMU positioned on the wither and from the kinematic marker fixed on the center of this IMU, the peaks corresponding to the synchronization keystrokes made at the start and end of recording were detected. Data were cut down to keep only the data between these two peaks on each of the systems. A correlation calculation was performed from the vertical position of the wither from the kinematic data and the IMU data to ensure that the data of the two systems were well synchronized. The average correlation value for all the trials of the eight horses is 0.84 ± 0.09.

Third, the locomotion parameters calculated from the MoCap were used to define the beginning and the end of the stance phase for each recorded stride. These events labeled respectively MoCapFootOn(*i*) and MoCapFootOff(*i*) of *i*-th gait cycle, were calculated using the method validated by Merkens and Schamhardt [22], with *i* equal 1 to n and n > 25. The stride duration named MoCapStrideDuration(*i*) (Equation (1)) and the stance phase duration MoCapStanceDuration(*i*) (Equation (2)) of the the *i*-th gait cycle were computed with MoCapFootOn(*i*) and MoCapFootOff(*i*) frames.

MoCapStrideDuration(*i*) = MoCapFootOn(*i* +1)-MoCapFootOn(*i*) (1)

MoCapStanceDuration(*i*) = MoCapFootOff(*i*)-MoCapFootOn(*i*) (2)

**Figure 3:**
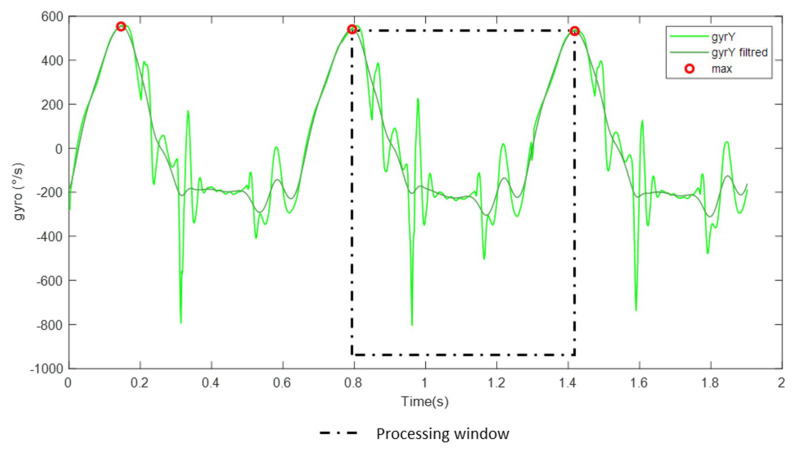
Representation of the Y-axis gyroscopic filtered signal used for the pre-segmentation of processing windows (**o**). In this figure, the *i*-th ImuWindow is shown in dotted lines. It is preceded by the (*i*-1) th ImuWindows delimited by the first two maximum points represented in red.

The fourth step was the data processing of the IMU data which included 2 substeps. The first substep was the windowing of the IMU data. From the low pass filtered signal (2nd-order lowpass Butterworth filter with a cutoff frequency of 20 Hz) of the Y-axis of the gyroscope, each maximum peak was identified (Figure 3). This filter eased to identify the cycle pattern of the gait cycle as we noticed that the average stride frequency for all horses participating in the experiment at 4 m/s is less than 10 Hz. Then, a processing window was determined between two consecutive peaks. Each processing window includes unique gait cycle data. So, *n* windows were identified and the *i*-th was named ImuWindow(*i*). 

**Figure 4:**
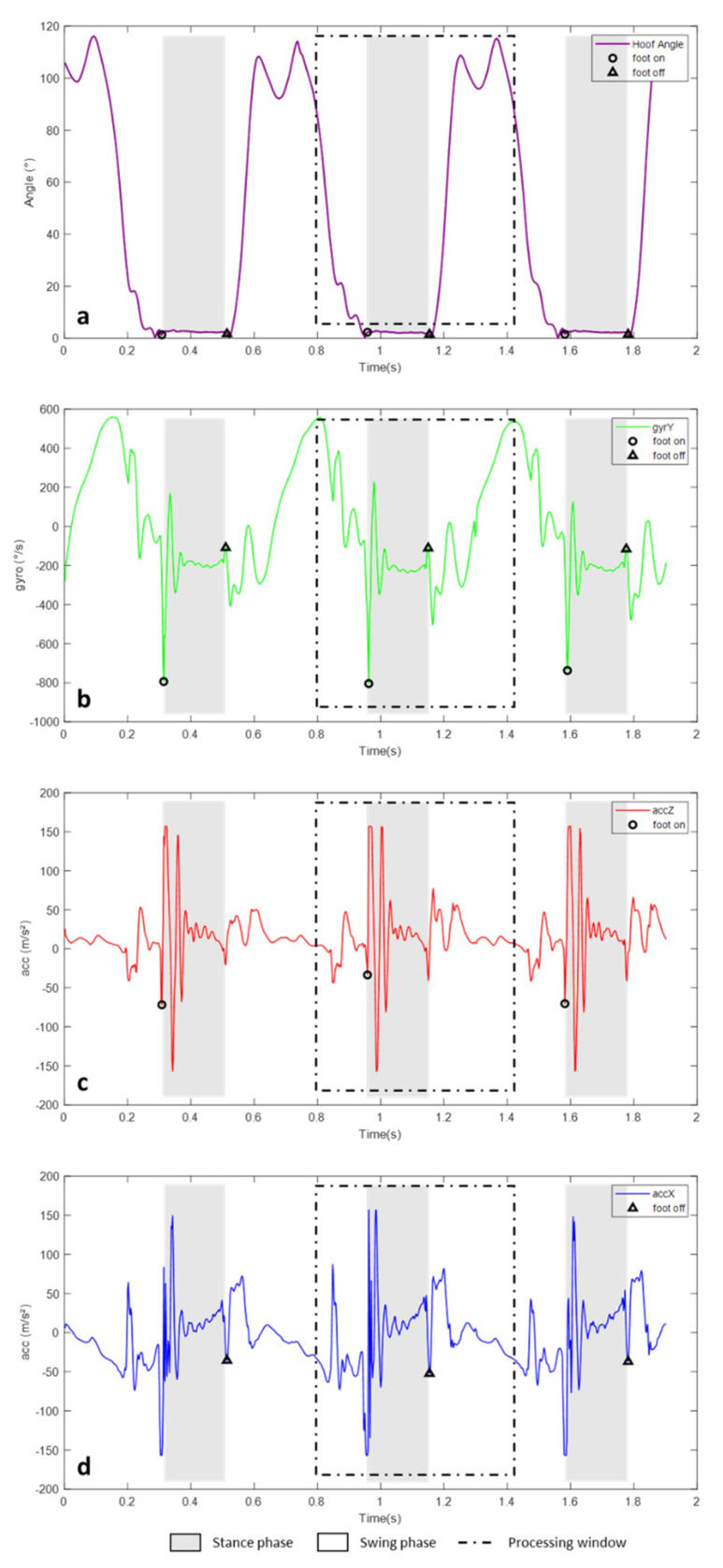
Representation of (**a**) the hoof angle calculated from the hoof markers allowing the detection of *Foot on* (o) and *Foot off* (**∆**) reference events (*MoCapFootOn* and *MoCapFootOff*), (**b**) the Y-axis gyroscopic signal used for the detection of *Foot on* (o) and *Foot off* (**∆**) events in method A (*ImuFootOn_A* and *ImuFootOff_A*) and method C (*ImuFootOn_C* and *ImuFootOff_C*), (**c**) the Z-axis accelerometric signal used for detection of *Foot on* (o) events in method B (*ImuFootOn_B*) and method D (*ImuFootOn_D*), (**d**) the X-axis accelerometric signal used for detection of *Foot off* (**∆**) events in method B (*ImuFootOff_B*) and method D (*ImuFootOff_D*).

The second substep was the detection of *Foot on* and *Foot off* events from IMU data by four methods. These events identified for each of the methods will then be compared with those detected on the MOCAP data in order to assess their accuracy (Figure 4a).

**Method A:** in ImuWindow(*i*), the first minimum peak on the Y-axis of the gyroscope was identified, corresponding to the *Foot on* event labeled ImuFootOn_A(*i*). Then for the *Foot off* labeled ImuFootOff_A(*i*) was identified as the peak preceding the penultimate minimum peak, detected with a threshold of 20% of the minimum value of the gyroscope signal on the Y-axis in the processing window (Figure 4b). 

**Method B:** in ImuWindow(*i*), on the Z-axis of the accelerometer, the *Foot on* labeled ImuFootOn_B(*i*) was identified by the detection of the minimum peak preceding the first peak max (Figure 4c). For the detection of the end of the stance phase, the jerk of the acceleration on the X-axis was calculated. This step detects the ‘plateau’ corresponding to the low-amplitude acceleration of the IMU during the stance phase. The end of the stance phase was then identified as the minimum peak on the X acceleration following the end of the plateau. This minimum peak corresponding to the *Foot off* was labeled ImuFootOff_B(*i*) (Figure 4d). 

**Method C:** The detection of the *Foot on* was done by a discrete wavelet analysis (DWT) of the Y-axis of the gyroscopic signal, performed using a fourth-order Coiflet as the mother wavelet and five levels of decomposition (coif4 level 5) [34]. The peak of interest was then sought on the reconstructed signal, which corresponds to the first minimum peak in each ImuWindow(*i*). Then, on the gyroscopic signal on the Y-axis, the minimum peak, closest to the peak of interest detected on the signal reconstructed in wavelets, was detected. This peak corresponding to the *Foot on* was labeled ImuFootOn_C(*i*). The *Foot off* was identified with the detection of the peak preceding the penultimate minimum peak, detected with a threshold of 20% of the minimum value on the gyroscopic Y-axis, in the processing window. The *Foot off* was labeled ImuFootOff_C(*i*) (Figure 4b). 

**Method D:** A DWT of the Z-axis of the accelerometric signal (coif 3 level 5) was performed for the detection of the *Foot on*. In each ImuWindow(*i*), the minimum peak on the reconstructed signal was detected. Then, on the accelerometric signal on the Z-axis, the maximum peak was detected preceding the minimum peak detected on the signal reconstructed in wavelets. The *Foot on* corresponding to the minimum peak preceding the first maximum peak on Z accelerometric signal was labeled ImuFootOn_D(*i*) (Figure 3c). A DWT of the X-axis of the accelerometric signal (coif 3 level 5) was performed for the detection of the *Foot off*. In each ImuWindow(*i*), the peak of interest corresponding to the end of the stance phase was detected on the reconstructed signal in absolute value. Then, on the accelerometric signal on the X-axis, the minimum peak, closest to the peak of interest detected on the signal reconstructed in wavelets, was detected. This minimum peak was labeled ImuFootOff_D(*i*) (Figure 4d).

The rationale for the use of wavelet reconstruction is due to its ability to perform a pattern-oriented analysis [35]. In Methods C and D, the selection of the type of mother wavelet Coiflets is performed by resemblance process [36].

For each method X = {A, B, C, D}, stride duration is named ImuStrideDuration_X(*i*) (Equation (3)), and the stance phase duration ImuStanceDuration_X(*i*) (Equation (4)) of the *i*-th gait cycle was calculated.

ImuStrideDuration_X(*i*) = ImuFootOn_X(*i* +1)-ImuFootOn_X(*i*) (3)

ImuStanceDuration_X(*i*) = ImuFootOff_X(*i*)-ImuFootOn_X(*i*) (4)

The results of each method were then compared to MoCap data to assess their accuracy and repeatability. The accuracy of *Foot on*, *Foot off,* as well as the *Stride Duration* and the *Stance Duration* of each method X= {A, B, C, D} compared to those measured on the data of the MoCap, was studied with Bland-Altman representation [37]. For each method, the accuracy was defined by the means difference (Bias) between the method values and the MoCap values and precisions as the standard deviation of the differences (SD). The limits of agreement corresponding to the confidence interval where 95% of the differences were represented were calculated [37]. The bias and the SD were used to estimate this interval (Equations (7) and (8)). For the results of *Foot on* and *Foot off* events, time was expressed as a percentage of the stride duration. The difference between the method measured and the MoCap was related to the duration of the corresponding stride measured on the MoCap data. The results of *Stance Duration* and *Stride Duration* were expressed by the difference in milliseconds between the duration measured from the *Foot on* and *Foot off* of the method used and those determined with the MoCap.

AgreementLimitHigh_X = Bias + 1.96xSD (5)

AgreementLimitLow_X = Bias − 1.96xSD(6)

## 3. Results

### 3.1. Foot on Detections

**Figure 5:**
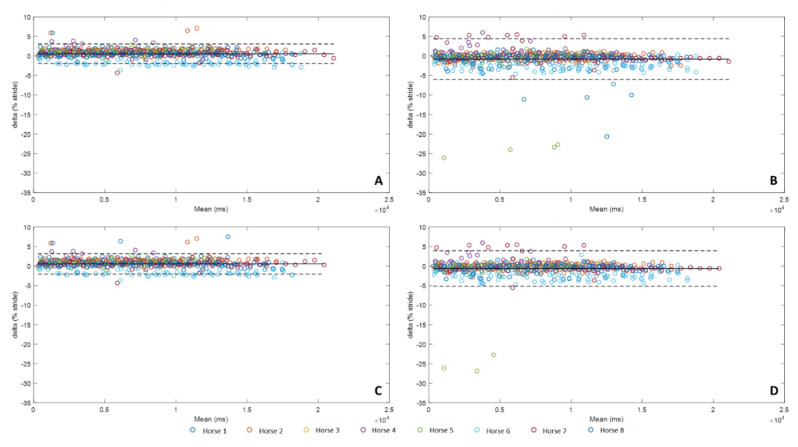
Bland-Altman comparison of the *Foot on* detection of the four methods developed with IMU data and MoCap *Foot on* detection. Accuracy (bias between each method and MoCap) and limits of agreement (95% limits of agreement) of method A were represented on the upper left corner (**A**), method B on the upper right corner (**B**), method C on the lower left corner (**C**), and method D on the lower right corner (**D**).

The following results present the precision of each method for detecting *Foot on* (Figure 5) in the percentage of stride.

For the detection of *Foot on* (Figure 5), whatever the method, the average bias was less than 1% of stride duration. The methods A and C show a tendency to detect *Foot on* with a slight delay, respectively 0.55% and 0.57%. Conversely, the methods B and D showed a tendency to detect *Foot on* slightly ahead, respectively −0.84% and −0.62%. The confidence interval was wider for methods B and D than for methods A and C (B: [−6.06%, 4.38%], D: [−5.17%, 3.92%] vs. A: [−1.95 %, 3.06%], C: [−2.05%, 3.19%]).

### 3.2. Foot Off Detections

**Figure 6:**
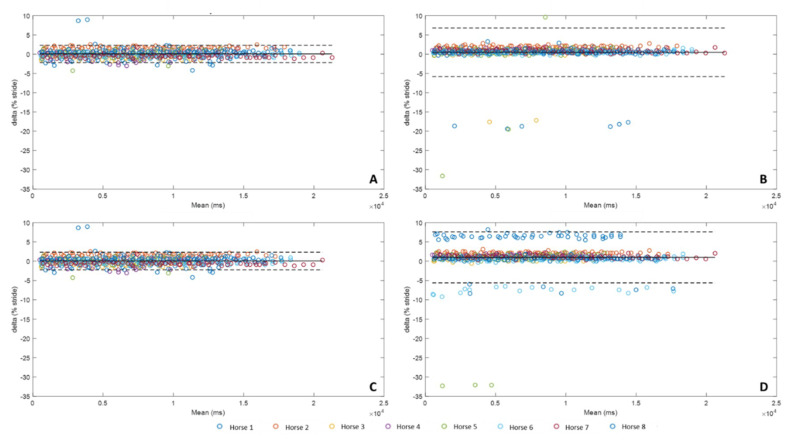
Bland-Altman comparison of the *Foot off* detection of the four methods developed with IMU data and MoCap *Foot off* detection. Accuracy (bias between each method and MoCap) and limits of agreement (95% limits of agreement) of method A were represented on the upper left corner (**A**), method B on the upper right corner (**B**), method C on the lower left corner (**C**), and method D on the lower right corner (**D**).

The following results present the precision of each method for detecting *Foot off* (Figure 6) in the percentage of stride.

For the detection of *Foot off* (Figure 6), Methods A and C presented an average bias of 0.05% while methods B and D have a higher bias, respectively 0.48% and 1.00%. The confidence interval was also very close for methods A and C (A: [−2.21%, 2.32%], C: [−2.23%, 2.33%]) and more extended for methods B and D (B: [−5.84%, 6.80%], D: [−5.62%, 7.62%]). For horses 1 and 6, a systematic detection error occurred with method D. For horse 1, method D detected the peak preceding the peak corresponding to the *Foot off* event. For horse 6, method D detected the peak following the peak corresponding to the *Foot off* event.

### 3.3. Stride Durations

**Figure 7:**
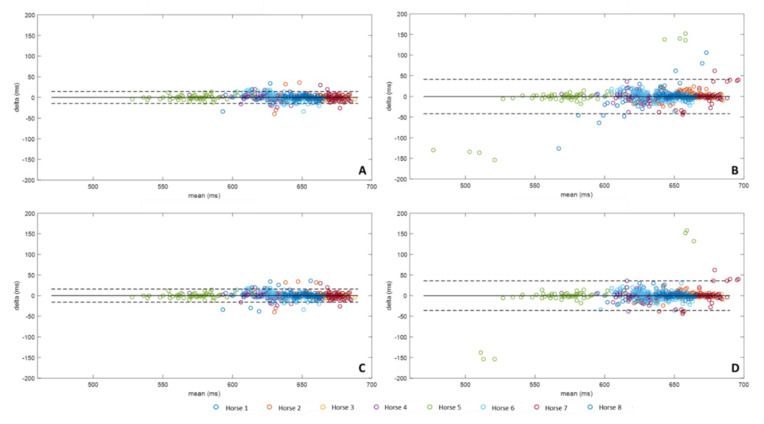
Bland-Altman comparison of the *Stride Duration*, calculated from the *Foot on* obtained from the four methods developed with IMU data and MoCap. Accuracy (bias between each method and MoCap) and limits of agreement (95% limits of agreement) of method A were represented on the upper left corner (**A**), method B on the upper right corner (**B**), method C on the lower left corner (**C**), and method D on the lower right corner (**D**).

*Stride duration* (Figure 7) was calculated for each method from *Foot on* according to Equation (3) and compared to *Stride durations* calculated from MoCap data according to Equation (5). The bias of each method was expressed in milliseconds (ms). All of the methods tested had a bias of less than 1 ms (A: −0.01 ms, B: −0.19 ms, C: 0.05 ms, D: −0.12 ms). However, methods B and D had a wider confidence interval than methods A and C, with B: [−41.76 ms, 41.37 ms] and D: [−36.09 ms, 35.84 ms], vs. A: [−14.52 ms, 14.50 ms], and C: [−15.74 ms, 15.83 ms].

### 3.4. Stance Durations

**Figure 8:**
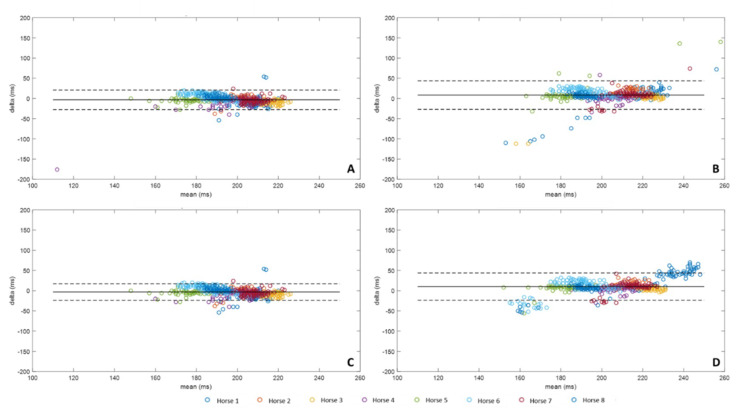
Bland-Altman comparison of the *Stance Duration*, calculated from the *Foot on* and *Foot off* obtained from the four methods developed with IMU data and MoCap. Accuracy (bias between each method and MoCap) and limits of agreement (95% limits of agreement) of method A were represented on the upper left corner (**A**), method B on the upper right corner (**B**), method C on the lower left corner (**C**), and method D on the lower right corner (**D**).

*Stance duration* (Figure 8) was calculated from the *Foot on* and *Foot off* for each method according to Equation (4) and compared to *Stance durations* calculated from the MoCap data according to Equation (6). Methods A and C had slightly underestimated *Stance durations* with a respective bias of −3.46 ms and −0.32 ms, while methods B and D presented overestimated *Stance durations* with a respective bias of 8.26 ms and 10.08 ms. The most restricted confidence interval was obtained with the method C (C: [−23.92 ms, 17.28 ms] vs. A: [−27.53 ms, 20.62 ms], B: [−26.89 ms, 43.42 ms] and D: [−23.56 ms, 43.71 ms]). As previously for *Foot off* detection, a systematic error was noticed for the horses 1 and 6 in *Stance Duration* with method D.

## 4. Discussion

The force plate and MoCap are the reference tools for the detection of stance phases [24,25,26]. Although these tools provide great precision in the measurements made, they can be used only in laboratory conditions which could be painstaking for longitudinal investigation, and exclude most of the field investigations. To avoid these limitations, several studies had developed new methods for the detection of stride events. Some studies used accelerometers positioned on the hooves [29], others used several IMUs positioned on different anatomical points of the horse [27]. This study opted for a single inertial sensor located on the lower limb. This choice was motivated by the versatility and the simplicity of the location and attachment. Indeed, IMU can easily be integrated into boots, the usual equipment of the horse.

In this study, four methods of detecting *Foot on* and *Foot off* of the horse locomotion by a single IMU located on the canon bone were compared. Two methods processed gyroscopic data of the IMU, using thresholding (method A) or wavelet (method C), and had performances superior to those developed using thresholding (method B) or wavelet (method D) from accelerometric data. These methods demonstrated a bias less than 0.6% of stride for *Foot on* and 0.1% of stride for *Foot off*. The measurements of the *Stance* and *Stride durations* were also more precise for methods A and C. Several methods were proposed to detect locomotion events by IMU measurement. Raw data of the accelerometers and gyroscope were commonly used. Methods by threshold detection on accelerometric signals were the most common [13,14]. Gyroscopic signals are often used in the case of human locomotion investigations [14,15,16] but these methods are also known to drift over time [38]. In this study, methods A and C used gyroscopic signals. Moreover, to limit the risk of drift, the post-processing was performed in time windows around 1s to 2s [39]. On the other hand, wavelet decomposition allowed a new approach by the detection of events through pattern recognition [40,41,42] and has been tested on accelerometric signals for human locomotion [43,44]. In this study, the wavelet decomposition was adapted for horse locomotion signals and extended to post-processing of the gyroscopic signals. 

A previous study [31] also evaluated the accuracy of different algorithms for the detection of *Foot on* and *Foot off* to determine *Stride duration* using a force plate as the gold standard. The best accuracy for measuring the *Stance duration* was −11.6 ms with algorithm 3 developed in the study [31] for the front limb at a trot. In our study, the accuracy of the methods developed was between −3.32 ms for the most accurate (Method C) and 10.08 ms for the least accurate (Method D). The difference observed in the evaluation of the accuracy of the methods developed in each study can be explained by a loss of accuracy by the use of MoCap for the *Foot on* and *Foot off* measurements compared to the force plate [45]. Nevertheless, our more accurate method (Method C) showed a more restricted confidence interval than that of the most accurate method of the study [31] (respectively [−23.92 ms, 17.28 ms] vs. [−79.4 ms, 56.2 ms]). Although it was difficult to compare the accuracy of the methods developed in each study, due to two different reference systems, method C developed in our study provided better reproducibility in the measurement of the *Stance duration*. 

This study demonstrated that the performance of the four methods was variable. The methods based on the processing of the gyroscopic data (method A and C) presented better results than ones based on the processing of the accelerometer data (method B and D). The less accurate results of methods B and D were dependent on the horse and we noticed that these methods failed for two horses. An explanation could be that the high energy impact of the hoof on the ground leads to vibrations from the impact wave, which could increase the noise-signal ratio [46,47]. Methods A and C reported better results and similar performance. Similar observations were noticed in the case of human locomotion [48]. 

However, our study presents limitations. First, the stance detections from the IMU positioned on the forelimb cannon bone were compared to the stance detections made from the MoCap on the hoof [22]. The horse’s foot and cannon bone are connected by three joints, the distal interphalangeal joint, the proximal interphalangeal joint, and the metacarpophalangeal joint (fetlock joint) [21,49]. These joints have a damping role in the locomotion of the horse which can cause a temporal shift of the stride events between the hoof and the canon bone. The conformation of the horse’s limbs and hooves can have an effect on the stride pattern [50,51,52]. This parameter could be variable from one horse to another and lead to differences in stance detections from the IMU more or less close to those made from the hoof (amplitude of the confidence interval). Second, in this study, a treadmill was used in order to control the speed and the regularity of strides. In the field, the nature of the ground leads to a change in the stride pattern [4,53], and the range of the inertial sensor values. The stride cycle detection with wavelets seems more reliable because this method is focused on the shape of the curves themselves correlated to the stride pattern [40,54]. However, the application of this method to different paces and types of ground surfaces will nevertheless require the adjustment of the type of wavelets. 

## 5. Conclusions

This study made it possible to compare different methods of stance phases detections from an IMU positioned on the cannon bone. The methods developed from the gyroscope have shown more precision on all the horses measured. The use of wavelets to detect the stance phases from the gyroscopic signal reduces the impact of the stride pattern specific to each horse. Adapting this method to different conditions (gait and ground) could allow the development of a simplified tool for the analysis of horse locomotion in the field and for longitudinal analysis.
